# Mechanistic-enriched models: integrating transcription factor networks and metabolic deregulation in cancer

**DOI:** 10.1186/s12976-015-0012-3

**Published:** 2015-09-09

**Authors:** Enrique Hernández-Lemus, J. Mario Siqueiros-García

**Affiliations:** Computational Genomics, National Institute of Genomic Medicine, Periférico Sur 4809, México City, 14610 México; Center for Complexity Sciences, National Autonomous University of México, Ciudad Universitaria, México City, 04510 México; Laboratorio de Redes, Instituto de Investigaciones en Matemáticas Aplicadas y en Sistemas, Universidad Nacional Autónoma de México, Ciudad Universitaria, México City, 04510 México

**Keywords:** Data-driven approach, Hypothesis-driven approach, Systems biology, Cancer

## Abstract

**Background:**

In the present paper we will examine methodological frameworks to study complex genetic diseases (e.g. cancer) from the stand point of theoretical-computational biology combining both data-driven and hypothesis driven approaches. Our work focuses in the apparent counterpoint between two formal approaches to research in natural science: data- and hypothesis-driven inquiries. For a long time philosophers have recognized the mechanistic character of molecular biology explanations. On these grounds we suggest that hypothesis and data-driven approaches are not opposed to each other but that they may be integrated by the development of what we call *enriched mechanistic models*.

**Methods:**

We will elaborate around a case study from our laboratory that analyzed the relationship between transcriptional de-regulation of sets of genes that present both transcription factor and metabolic activity while at the same time have been associated with the presence of cancer. The way we do this is by analyzing structural, mechanistic and functional approaches to molecular level research in cancer biology. Emphasis will be given to data integration strategies to construct new explanations.

**Results:**

Such analysis has led us to present a mechanistic-enriched model of the phenomenon. Such model pointed out to the way in which regulatory and thermodynamical behavior of gene regulation networks may be analyzed by means of gene expression data obtained from genome-wide analysis experiments in RNA from biopsy-captured tissue. The foundations of the model are given by the laws of thermodynamics and chemical physics and the approach is an enriched version of a mechanistic explanation.

**Conclusion:**

After analyzing the way we studied the coupling of metabolic and transcriptional deregulation in breast cancer, we have concluded that one plausible strategy to integrate data driven and hypothesis driven approaches is by means of resorting to fundamental and well established laws of physics and chemistry since these provide a solid ground for assessment.

## Background

Traditionally biochemical and molecular biology studies have relied heavily on small –mechanistic– models in order to test hypothesis. A great deal of this way of working depended on the intuition and insight of leading experts on highly specialized subjects that define the lines of inquiry as well as the methods to be followed, mainly guided by their own knowledge and experiences [1, 2]. Since the advent of high throughput experimental techniques in genomics and proteomics, computational biologists, bioinformaticians and theoretical biophysicists have developed analytical tools for the automated, unsupervised analysis of huge amounts of biodata. Unlike the former case, these techniques are rooted in probabilistic modeling, machine learning and statistical significance to extract conclusions and is commonly argued that these are independent of the beliefs and conceptions of the investigators, that is to say, theory-free [3]. The former approach is usually termed *hypothesis-driven* and is conceived as bottom-up research whereas the latter investigations have been known as *data-driven* and are conceived as top-down.

In the last few years a debate about the possible taking-over of the data-driven approach and the imminence of the hypothesis-driven strategy obsolescence has emerged, particularly in the context of the Life Sciences. Many different points of view and perspectives have been developed by scientists and philosophers and are not limited to the dichotomy between those who support a data-driven approach and those who support a hypothesis-driven preference of doing science. Such perspectives represent deeper epistemological and sociological concerns regarding whether the notion of causality is still relevant, the role of models and simulations, the purpose and usefulness of hypothesis in science, the differences in *ways of knowing* as well as the differences in the cultures of doing science, and the very nature of the *Bio-disciplines* as Science or Engineering [1], and among them, being these approaches so different, how are we to reconcile them in an integrated frame.

The cornerstone on which the debate lies, is the question of whether data-driven or hypothesis-driven research are better suited for generating knowledge from the great amounts of data produced by powerful computers and algorithms that time and again keep on growing in power and performance [4]. From the persective of a scientist, the debate is about which is the best way to understand and explain a phenomenon, if it suffices to deal with biological phenomena exclusively from a data-driven perspective, or if this is not the case, how it is related to a hypothesis-driven strategy. Even more relevant is that integration of both strategies is the emerging trend, and the questions might be towards how is this integration taking place. Our approach is primarily centered on the the third question. Based on our own experience on Cancer genomics we will present how have we proceeded in integrating hypothesis-driven inqueries and *Big data*. In order to develop our ideas, we also take advantage of the work that philosophy of biology has already done on the subject.

### Integration

Systems Biology relies on two distinct approaches: a top-down, data-driven (DD) and a bottom-up, hypothesis-driven (HD) perspectives. Although these two approaches belong to such general frame, due to the great advancements in data-mining, sorting algorithms and super-computing in general, there is a hype exalting the data-driven perspective virtues, apparently overshadowing the more traditional, hypothesis and model based approach from molecular biology. This has trigger the alarm among scientists and philosophers who have suggested that an integration of top-down and bottom-up approaches is needed [1, 5, 6]. The current call for the study of *integration* doesn’t end with bringing HD and DD together, it also includes datasets integration, the integration of explanations and even the integration of disciplines.

From the perspective of Philosophy of Science, integration may be a much older subject of analysis. Integration is a notion that might seem close to the idea of the unity of science, an idea that states that concepts and laws can be reduced to the most fundamental elements [7]. In the context of Biology, philosophers have neglected the applicability of such reducionistic view portayed by the ideal of the unity of science. Instead, they have endorsed a pluralistic perspective regarding explanations. Philosophers of biology consider that statistical and causal-mechanistic explanations are the most common. Statistical explanations may be found in disciplines such as population genetics and evolutionary biology. On the other side, explanations based on the idea of mechanisms belong, for instance, to molecular biology [8–11]. Philosophers of biology are aware of the diversity and uses of different strategies in generating biological explanations for which, integration is not so much perceived as a way of unifying or reducing biological explanations but as a way of bringing together entities and practices performed by biologists [12, 13].

Among the most recent and interesting work regarding integration is the one of Sabina Leonelli. Leonelli has studied the problem of data classification, vocabulary development and bio-ontologies, as well as the problem of datasets integration as an epistemic issue. On datasets integration she has analysed the case of plant biology to three different levels: 1) inter-level integration in which data belonging to the same species but to different levels of description are brought together in order to gain interdisciplinary and holistic knowledge about the organism; 2) Cross-species data integration, which aims to obtain knowledge about different manifestations of the biology of different species; 3) Translational data integration that has the purpose to obtain knowledge towards finding solutions to societal problems [14–16]. Leonelli demonstrates that infrastrcuture and standarization involved in data-integration plays a crucial role in generating new knowldge. She has also shown how epistemic goals lead the process of integration producing different structural configurations of data.

Besides data integration, authors like O’Malley and Soyer have studied methods integration and explanations integration in the context of Data-driven and Hypothesis-driven research [12]. O’Malley and Soyer have suggested that despite the classificatory virtues brought by the distinction between Data-driven and Hypothesis-driven methodological approaches, it is too broad to be of any analytical use. Instead these authors suggest other strategies in order to achieve a better understanding of the dynamics of contemporary biology and their practices. Such strategies have to do with how different methodologies are combined in order to gain any knowledge about one biological system that otherwise cannot be obtained using a sigle method. According to these authors, integration of methods in Systems Biology may imply the use of iteration in two different ways, one of them is the simple iteration of a set of methods through which a “refinement of the model by hypothesis testing” is achieved. The other one requires the iteration of the products of DD and HD so to examine a biological domain or phenomenon [17]. The analyses of data integration are also part of the strategies aforementioned. Data integration makes reference to the use of technical and theoretical processes in order to harmonize datasets in a way that they can be brought together and maximize their re-usability and re-analysis. One final strategy is explanatory integration. Integration at this level may be conceived as the use of techniques and means to bring together different models into a specific area of inquiry along with their respective explanatory resources.

The kind of integration that is closer to our aims and views is the one studied by Ingo Brigandt. Brigandt has explored the role of mathematical explanations and its integration with mechanistic explanations in the context of Systems Biology. On this regard, we believe that there are two important contributions of his work to the understanding of how explanations are being developed in contemporary biology. Philosophical accounts on what is a mechanistic model are prone to think about them as a static structure. Brigandt, in the same vein as William Bechtel, have added to the notion of mechanisms a dynamical character. Such dynamical property is called by Bechtel *functional dynamics*. Once dynamics are introduced to the concept of mechanism, mathematical explanations have a place in the global explanation of the phenomenon (still explained in causal-mechanistic terms). Dynamics becomes the open door in order to integrate mathematical and mechanistic explanations. Integration takes place when the *explanatory relevance* of mathematical explanations or *ER* as Brigandt calls it, is recognized as a component that adds explanatory power to the mechanistic model. When mathematics are introduced to causal-mechanistic models, a quantitative dimension complements the model that is described in terms of qualitative interactions. The quantitative complementarity to the qualitative model is explanatory relevant because certain knowledge is gained due to its presence, that otherwise would not be possible.

In this paper we argue in favor of an integration. It is true that the idea of integrating DD and HD is a recurrent theme in Systems Biology [6, 18]. But along with O’Malley and Soyer, we believe that the distinction between DD and HD is simply too broad for being useful. From our point of view, the integration of the two main approaches in Systems Biology could proceed as a process that would lead to the development of *enriched mechanistic models*. Enrichment implies two things: the first one is a hierarchical perspective. As it has been already pointed out by others, Systems Biology and complex biological systems imply a multilevel organization [12, 19–22]. In this guise, we draw the idea from our own experience that mechanistic models that describe the molecular relevant parts and their qualitative interactions (such as transcription factor networks and pathways, in which the chain of causal events between genes are stated), can be enhanced in a meaningful way by adding certain porperties from the level below (e.g., physico-chemical properties that determine local interactions at the molecular component level) and by adding to the model the constraints derived from the level above (e.g., the set of emergent patterns regarding transcription factors networks from the analyses of a gene expression dataset). Second, any attempt to add a component to the mechanistic model from the level above or below has to be *explanatory relevant* as Ingo Brigandt has suggested [13, 19]. Proceeding in such fashion, DD and HD distinction can be avoided but the products of their implementation can be successfully integrated. Finally, our goal in this paper is present how we believe a model for transcriptional regulation coupled with metabolism in cancer development may display important features of how mechanistic models are genetated by integrating different entities and practices in Systems Biology.

Our paper is structured as follows: in section two we present a brief summary regarding how researchers have conceptualized cancer. Section three introduces the idea that physicochemical modeling might be a well suited tool for integrating the former approaches as used in biology. In section four we present our account on integrating metabolism and molecular aspects on cancer by bringing together mechanistic models, data-driven transcription factor networks and cell-level energetics. Finally we present some general conclusions.

## Cancer and metabolism: a conundrum

Cancer is probably one of the best studied diseases. Historically, cancer has been studied from a hypothesis-driven strategy and explained in terms of causal-mechanistic models. Today, great amounts of data have been collected and processed about this disease (or better, set of diseases), making possible to do research from a data-driven approach.

It is a known fact that tumor cells display striking differences in their metabolic functions as compared to normal cells and often resort even to characteristic biochemical pathways to supply for their energetic requirements. Due to this, neoplastic tissues express tumor-specific enzymes belonging, in general, to the family of the glycolytic enzymes (GEs). GEs interact with other modulators of tumor behavior (TMs) in order to adapt their metabolic functioning to the extreme proliferative regime under hypoxic conditions typical of tumor tissue [23].

The analysis of the interplay of GEs and TMs have recently called the attention of oncology researchers since is hypothesized that inhibition of GEs or appropriate tuning of TMs may leave tumors out of energy, while leaving non-tumor cells unaffected. It seems thus, that therapeutic regulation of cancer-related energy production pathways may become a substantial research area for pharmacological therapy in cancer [24]. Due to selective advantages displayed by tumor cells, however, therapies must be applied cautiously in order not to annihilate normal cells along with neoplastic ones. In this regard, it has been discussed (in the context of pancreatic tumor cells) that a combination of agents that inhibit both energy production and cell signaling may lead to the development of multiplexed therapy to target malignant cells effectively [25]. In the following sections we will recount how cancer biology research has used different methodological HD and DD strategies throughout its history.

### The usual suspects: hypothesis-driven molecular oncology

Traditional thinking in cancer research has been focused in the role played by some quite specific molecules termed *oncogenes* and *tumor suppressors*, both of which types commonly present mutations in their associated DNA loci. These mutations may be single base changes, regional variations leading to copy number variants on their genes and even chromosomal rearrangements associated with *fusion genes* or *chimeric proteins*. All of these phenomena have been generalistically termed *genome instabilities* when they are related to the onset and development of cancer [26].

Under this view, cancer is produced by the action of *oncogenes* (OG) -defined broadly as *genes with the potential to cause cancer*- that are frequently either mutated, over-expressed or both, in cancer cells in comparison to non-tumor cells [27]. Once these genes are on, tradition says, cancer will appear. In the other hand there are the *tumor suppressor genes* (TSG) that are, of course, *molecules that protect the cells from cancer*. If tumor suppressors are mutated in such a way that there is a loss or reduction in its function, the cells may become neoplastic [28]. Under this paradigm (presented here in an extremely over-simplified version), cancer research may be focused in finding such oncogenes and tumor suppressors (and of course, every possible combination of them) and then looking up for ways to disassemble the former and improving the latter, and make them work together in order to reverse or avoid carcinogenesis.

Of course, there is much more involved in the OG/TSG theory of carcinogenesis. However, even the refined versions of the theory face important challenges that have led to a broadening of the scope of cancer research in recent times. The usual OG/TSG approach to cancer research consists in: (**version 1**) *finding* a molecule whose activity is abnormally high in malignant cells, *hypothesizing* that such molecule is an oncogene. *Looking for* other processes that *somehow* activate a formerly inactive form of the molecule (call that a proto-oncogene) and then studying the molecular interactions between the proto-oncogene and its activators. These interactions, along with the structure of the proto-oncogene and specially the activity of the oncogene *are the foundations of carcinogenesis*. Or (**version 2**) *finding* a molecule whose activity is repressed or absent in tumors, *hypothesizing* that such molecule is a tumor suppressor. Then *looking for* processes that inactivate the tumor suppressor (either by mutation or by other functional processes), such interactions and the structure and function of the tumor suppressor *determine the origins of cancer*. Or, more frequently, (**version 3**) a combination of several instances of versions 1 and 2, i.e., cancer appears *due to* the presence of a number of oncogenes activated by pro-malignant events that include a number of inactivated tumor suppressors.

Thus, according to OG/TSG theories there are a number of important molecules (and their associated pathways) that are the key players in cancer: Oncogenes such as RAS, MYC, EGFR, VEGFR, WNT, ERK, TRK, etc. Fusion oncoproteins such as BCR/ABL and tumor suppressors such as p53, BRCA, PTEN, CD95, and others. These are *the usual suspects*, whenever there is cancer, *these are the molecules that one should look for*.

The approach just sketched, although successful to an extent in unveiling *some* issues in cancer biology, is confronted with a number of problems. As is clearly exposed in the review by Hanahan and Weinberg [26], one of the identifying marks of cancer is *genomic instability*. Malignant cells often present extreme variations in their genomes, both at the sequence (large chromosomal rearrangements, disparate copy number variants, lots of mutations, etc.) and at the gene and protein expression levels (distinctive expression profiles, aberrant proteins, etc.). Amidst such a large number of abnormalities, is not that clear how can one distinguish between *driver events* and *passenger events*. To put it simple, there is no easy way to distinguish abnormalities that *cause* cancer from abnormalities that appear as a *consequence* of cancer. The onset of cancer is elusive, *unless* you actually *take action to initiate* cancer (say in an animal model) but then your observations will be necessarily biased.

Another example is the case of OG and TSG. The paradigmatic example of a TSG is *p53*, a gene that is either mutated, deleted or abnormally functioning in more than 50 % of human tumors. However, even if p53 has been extensively studied (possibly more than any other biomolecule) for many decades, there is still no substantial advancement in cancer prognostics and diagnostics based on its role alone. The reason is that while its is known that DNA damage mechanisms (in whose repair is involved p53) are fundamental in carcinogenesis, it is now clear [26]) that this is by no means an isolated process.

### The not-so usual suspects: data-driven quantitative analysis of high throughput experiments

Recent years have witnessed the rise of the data-driven approach for the study of complex diseases like cancer. This approach has been motivated both, by the overwhelming complexity of biological systems and by the technological breakthrough represented by high throughput (genome wide) genomic technologies and high computing data analysis capabilities. Under the data-driven view, a problem like cancer is approached by a thorough systematic genome wide study of cancer samples and controls. By analyzing the statistically significant differences in molecular profiles (DNA mutations, gene expression patterns, proteins, etc.) between cases and controls, usually combined with computational classification methods and database assessment (all of these, in principle, devoid of any preconceived hypothesis) one looks for molecules and pathways that may be relevant to cancer (or any other disease or phenotypic condition).

Interestingly enough, high throughput data-driven approaches have identified molecules and pathways that, though relevant to cancer biology, do not belong to either the OG or TSG classifications. Moreover, many of such processes were previously not even considered related to cancer phenomenology. Such new targets have broadened our knowledge about the molecular origins of cancer, while, at the same time, have unveiled how little we knew (and still know) about the intricacies related to the origin and development of malignancy. Energetic deregulation at the cellular level, immune system adaptability, hypermutation pathways and genome instability, as well as abnormal inflammation processes have emerged as fundamental to understand the origins of cancer and not only (as it was previously believed) as unpleasant consequences of malignancy [26]. Other processes such as the ones related to aging, rescue from apoptosis and autophagia to mild proliferative states (that may later become highly proliferative such as those in cancer) and others, that are not yet established as *hallmarks of cancer* are also being found more and more often to be relevant to the neoplastic condition in a series of data-driven studies.

The particular case of the interplay between metabolic deregulation and gene expression instabilities has been attracting attention recently [23, 24, 29]. For a long time, the role that metabolic abnormalities may play in tumorigenesis was highly overlooked. It was considered that large scale metabolic changes were a consequence of tumor growth (which of course they are) with no involvement in the origins of neoplasia, something that is less and less considered to be true.

Data-driven approaches are not free from shortcomings. Arguably, the strongest limitation of the data-driven view is that, even under stringent statistical significance bounds, it is extremely difficult to ascertain whether a discovery is providing a genuine hint that may help us disentangle the complexity of disease or it is a *false positive finding*, an unavoidable question of our dealing with such large amounts of experimental data, relatively small sample spaces (orders of magnitude smaller in number than corresponding parameter and variable spaces) and biased annotation databases.

Large amounts of experimental data mean that, by chance alone, one may be able to find a number of results that *seem to be* correct and even repeatable but are not *real*. Since we are dealing with this, under the so-called *dimensionality problem* (i.e. a much lower number of experimental samples than the number of variables) it is not possible to overcome false positives by stringent re-sampling (all the contrary). Biased databases imply that one may find a true positive signal only to later discard it due to incomplete (or incorrect) annotations. Under this light, even data-driven approaches are indeed not *free from hypothesis*: which statistical or computational method we use and why, how we determine thresholds, bounds and other *free parameters*, which database we use to look up for; all of these are assumptions that are not provided by the data but, again, depend on the individual (thus limited) capabilities of the PI or researcher.

Data driven approaches have revealed important genes and pathways in cancer. There are a couple of well known examples. One of them is the discovery of computationally infered *gene expression signatures* that have unveiled important features like breast cancer subtypes [30, 31] and other tumors’ classification patterns [32]. Data-driven approaches have also led to the discovery of master regulators in B-cell malignancies [33] and breast cancer onset [29] and metastasis [34].

The second one is related to the construction and assessment of systematic strategies for the meaningful computational analysis of high throughput data. Worth mentioning is the role that *crowdsourcing* efforts may play. One of such crowdsourcing initiatives is the *DREAM Project* [35] developed by a core of computational and Systems Biology researchers at IBM’s Computational Biology Center and Columbia University [36].

The goal of the DREAM project is to *create a formal self-assessment methodology in which models in particular networks of interactions are inferred from data with a more quantitative sense of the accuracy of the predictions* by means of *challeng[ing] researchers in the field to perform predictions blindly on networks that have already been accurately mapped and validated a so-called gold standard network but which are known to only a few evaluators who do not participate in the analysis* [36]. The DREAM initiative has been quite successful by focusing on one or two different challenges each year for the last six years. A similar approach may be find in the CAMDA project [37] initially focused on techniques for DNA microarray data analysis and now broadened in scope to all kinds of massive biological data.

### Network addiction: a challenge

In the genomic literature there is still a reminiscence of classical genetics that lead us to perform genome wide analyses while ending up discussing single gene issues or at most talking about pathways involving individual molecules. This is of course changing: gene interaction networks are more and more at the center of discussions on cancer phenomenology. Researchers are realizing that the role of gene regulatory networks it is even more important than individual gene contributions [38]. In the past, molecular oncologists argued about *oncogene addiction* claiming that despite cancer’s complexity, the growth and survival of tumor cells may often be strongly limited by the inactivation of a single oncogene [39] that may in turn provide for a rational target for molecular therapy. More recent studies, however point-out to a more complex scenario in which tumors are not *addicted* to a single molecule but rather to the action of specific pathways and even whole networks in a phenomenon that has been called *network addiction* [38].

This shift of emphasis is way more than a semantic or cosmetic change, since it implies a radically new way of looking at the molecular origins of disease. Under the network addiction framework, different components of a cancer network may be deregulated thus affecting the biochemical dynamics of the entire network and, ultimately, those of whole cells and even populations of cells. We have already discussed the computational complexity involved in the inference of genome-level GRNs focusing on high throughput molecular data. Additional complexities arise due to the combinatorial nature of the different sub-networks or sub-pathways into which a genome-wide network can be decomposed to look up for their *oncogenic features* playing the role of complex analogs of OG and TSG.

Having already considered the challenges faced by high throughput data-driven approaches, quite especially those related to intelligibility and *feature selection* (i.e. how to construct meaningful models from high throughput data), the problem of analyzing cancer biology from the stand point of the network addiction paradigm may seem very hard to tackle. In reference [29], we used an integrative approach based on the combination of several instances of data-driven discovery and several instances of hypothesis driven modelization, aimed at providing some clues as to the role played (via network-level pathway *crosstalk*) by metabolic deregulation in transcriptional instability associated with primary breast cancer onset. The integrative approach used there, was firmly based in both kinds of views (data- and hypothesis- driven), unified in the context of a non-equilibrium thermodynamics framework. In the next section we will present some of the main ideas behind such approach.

## Results

After careful reflection on the way methods and approaches both from data-driven and hypothesis-driven research were integrated in the referred work in the interaction between metabolic deregulation and abnormal transcription patterns in breast cancer [29]. The main meta-methodological results may be summarized as establishing a solid, well-founded theoretical framework (in this case based in laws of thermodynamics and chemical physics) and using them to build mechanistic enriched models. We will elaborate in these results in what follows.

### Physicochemical modeling provides one theoretical framework to integrate omics and computational Biology studies

In general, Computational Biology studies are rooted in the search for functional patterns aimed at a statistical and/or probabilistic description. In contrast, part-based studies, such as those present in experimental Molecular Biology tend to be more focused in the interaction of the components leading to such functional patterns. From this perspective, cancer biology is perceived as a more *phenomenological* framework. In some sense, the situation is analog to what happens in thermal physics: there is a phenomenological representation or characterization given by thermodynamics, but there is also a probabilistic description in terms of microscopic states as given by statistical mechanics. In the end, both view points complement each other to the point that, in practice, they have become together one undivided discipline. Statistical mechanics provides thermodynamics with models and tools for calculation that allow for deeper understanding; while Thermodynamics provides context and a systematic framework that bound and regulate the theoretical representation models. Both views are unified in a formal structure: the laws of chemical physics.

Physicochemical models thus allow the unification of the microscopic and macroscopic views of matter. In reference [29] we thus resort to physicochemical modelization (under the tenets of non-equilibrium thermodynamics) as a means to unify the views of data-driven Computational Biology and hypothesis-driven cancer (molecular) Biology. The rationale behind was that a series of studies rooted in thermodynamics have already highlighted the fact that cell energetics may be playing a non-trivial role in the onset and development of malignancy [40–43]. Cellular communication processes (such as those regulating biochemical pathways) often rely on free energy transduction mechanisms [44]. Free energy distributions inside the cells hence determine to an extent which biomolecular processes are active and which are not, thus providing organisms with regulatory *locks and triggers* to control their functions. Abnormal energy profiles (such as the ones present when cells are under metabolic deregulation) may allow for *un-intended* biomolecular processes to happen. Under very special occasions, these processes may induce cascades or avalanches of unexpected events driving the cells unto uncontrolled states such as those found in carcinogenesis. These ideas are, of course not new [45] but they have acquired new life in view of current *genome-scale* analyses of biological phenomena [46].

In the particular case discussed in reference [29], we investigate the connection between energetic deregulation at the level of cell metabolism and global transcriptional instability triggered by master regulators. Master regulators (MRs) are transcription factors hierarchically located at (or near) the top of the cells’ transcriptional *programme*, i.e., are transcription factors for which a number of their targets are also transcription factors (even other MRs). The action of MRs may thus be able to trigger transcriptional cascades in which a big number and variety of mRNA transcripts are synthesized (transcribed) in a series of connected events. MR cascading phenomena is important for certain stances in cell development, differentiation and proliferation, but their inappropriate function could lead the cells into neoplastic states [33, 34]. We find out that non-equilibrium free energies provide a realistic description of transcription factor activation in the case of MRs. By studying their behavior at the gene regulatory networks level, we can systematically find deregulated pathways. Since most deregulated pathways found there were important in cancer biology, that work provided us with hints towards *a novel potential role of transcription factor energetics at the onset of primary tumor development* [29].

Wrapping-up, the emergence of statistical mechanics lead us to suggest a hierarchical way of thinking about integration. Just as thermodynamics and statistical mechanics refer to different description levels, so it is for us the network and moleculular energetic description levels as already mentioned in the paragraphs above. In what follows, we suggest that integration of these two levels can be integrated as part of one middle ground: the mechanistic models of Molecular Biology.

### Mechanistic models enrichment: cancer transcriptional networks meet metabolism

The concept of mechanism is one that has been well developed by philosophers. One may find different perspectives on what a mechanism is in the context of Biology, and particularly in the case of Molecular Biology. In general terms, mechanisms include several entities and their causal interactions, which may be physical or chemical in kind.

Some definitions are the following:

Mechanisms are entities and activities organized such that they are productive of regular changes from start or set-up to finish or termination conditions [47].

...a structure performing a function in virtue of it component parts, component operations, and their organization. The orchestrated functioning of the mechanism is responsible for one or more phenomena [48].

A mechanism is temporally extended, where some of the entities involved change their positions or their properties as a result of the mechanism’s action. [...]. What is explained [by a mechanistic explanation] is the outcome state of a token mechanism, or the behavior that is regularly produced by a type of mechanism ([13], p.74).

As it can be inferred from a previous section of this manuscript (section 2.1), cancer explained in terms of oncogenes and tumor supressors is remakably mechanicistic. Data driven research and network-based accounts –which may count as a special case of data-driven methods– are not that different since its main goal is to identify *parts* and significant *interactions* –global interactions. Data-driven research (including network analysis) is fundamentally a probabilistic enterprise, nevertheless, its aims are meant to massively identify mechanistic patterns. In other words, the products resulting from Data-driven research are not devoided of mechanistic meaning.

Along with such general notions of mechanism, we believe that it is necessary a hierarchical modeling approach in order to obtain integration through a process of *model enrichment*. The idea of hierarchical integration can be traced back to the second quarter of the 20 ^*th*^ century. Levels integration was the central idea of *Organicism* and the *Theory of integrative levels* (from now on TIL) and it was developed in order to study biological complexity. These approaches were a response to reductionism –to which they shared a materialistic philosophy– but they were different in their ontology and epistemology because they suggested that biological phenomena cannot be reduced to its basic components. Instead, it was proposed that for being able to harness biological complexity, a multilevel perspective is needed [21, 49–51]. In a similar way, we believe that the study of different levels of organization may display different modeling and explanatory strategies according to particular level properties and disciplinary traditions. We believe that such is the case when a genetic transcription mechanism is studied from their energetics, in which what we are seeing is not the mechanism but the energetics that regulate the interaction of its components, or when it is studied from a network perspective that allows to see other similar –newly discovered– mechanisms, parts and interactions.

The integrative analysis presented in reference [29] will serve as an example of *symbiotic* (so to speak) coexistence of data-driven and hypothesis-driven approaches. Integration takes place in our example by means of bringing together tools that are commonly applied to different levels of organization (*sensu* Organicism and TIL) to one class of mechanistic models of transcription factors pathways. Integration, in these terms is what we call *model enrichment*. Such a study starts with the analysis of a set of experimental data coming from 1191 whole genome gene expression experiments. By means of statistical analysis, we determined a set of differentially expressed genes between cases and controls. Once we had the set of significantly expressed genes, we proceeded to mine the data (the list of differentially expressed genes) and mine databases looking for both transcription factor activity and metabolic activity. This step implied the computational mining of databases from a large list of somewhat undisclosed molecules. These first activities were completly devoted to the identification of parts. The set of genes that were selected because it is assumed that are involved simultaneously in metabolic and transcription factor activies, are the ones that should become the components of the mechanistic model. Albeit there is a hypothesis regarding the connection between genes that play a role in metabolics and in transcription, data-mining makes possible to identify parts and interactions that are not limited to a unique transcription/metabolic mechanism. It is more the case that mining big data will inform us not just about one mechanism but also about many others that may respond to similar principles.

We looked for metabolic and transcription factor activities assuming that there was a role played in the coupling mechanisms at the molecular level. This required us to mine for thermodynamic parameters of such molecules. We obtained data about the free-energies of formation of the recently disclosed molecules. A specific non-equilibrium thermodynamical model and the data about the components free-energies, gave to our model a *bottom-up* meaning in two ways: first, the themodynamical model provided us a notion of what are the possible interactions between genes according to the system’s energetic and enthropic constraints. Second, free-energies are quantitative properties of the mechanism components that dictate plausible orderings of the parts and plausible routes for events to follow, in other words the direction of the mechanism dynamics. What we did by using this bottom-up approach, was adding to our model properties from the level underneath the mechanism in which we were interested, as well as the theoretical tools from that lower level.

Once we had the list of genes, transcription factors and energetics coming from the previous step, we performed data mining for associated biochemical pathways and protein-protein interactions, as well as probabilistic reconstruction of gene regulatory networks. By integrating both protein-protein interaction pathways and transcription factors networks it was possible to identify to what different pathways belong the set of genes and their products. We found that these pathways are deregulated (e.g., apoptosis, DNA repair, glycolisis), and that the cell has no alternative paths to compensante such deregulation. This part can be seen as an exploration of the level atop from which a global context for the parts of our model was defined. By these means, the parts of our model were placed –without a previous hypothesis– in their respective biological processes, but also it displayed how different components are active in different processes and how different processes may be interconnected.

Finally we built an integrative model that points out to transcription factors (TFs) as molecules whose expression is activated at low energies. This may be related with the fact that TFs are involved in the transcriptional activation of other genes. Hence, we can in principle expect that they are synthesized in primal stages when energy is started being released in the cells by metabolic processes. Transcriptional target genes (TGs) will be, in general synthesized later at higher activation energy levels. Still, within the group of TFs there may be some genes that are upstream in the transcriptional cascades, termed master regulators (MRs). Such MRs must present even lower activation energy barriers. By means of the combined approach just sketched, we were able to identify a small set of putative MRs that potentially are influencing the transcriptional cascades characterizing the differential gene expression profiles under primary breast cancer phenotypes. In the overall, the biological meaning of our example is that the model represents a mechanisms of *lock-in-the-trigger*, for transcriptional cascades.

## Conclusions

In this work we have discussed about the recent debate on whether Data-Driven and Hypothesis-driven approaches to science (in particular regarding Systems Biology) are meant to be juxtaposed or confronted with each other, or if one of these is intended to act as a counterpoint to the other, or in any case how are these two disimilar and apparently disparate views on science to be reconciled. This debate has been recently fueled up with the recent advent and promisory success of *big data science* that for some may point out to an upcoming disruption of hypothesis-driven approaches to scientific inquiry. Such a view, although not generalized at all shed some light on important matters such as the role of *causality* as is usually understood in the further development of science. We argue that this is not the case and that, in fact, Data-driven science is not *Hypothesis-free*, i.e. data driven research is not independent of the beliefs and views of the investigator, rather such hypoteses are immersed in the methods of inquiry used to deal with data.

In line with these thoughs (following several other scholars such as Brigandt and Bechtel, for instance) we wonder how integration is taking place in the emerging frame of Systems Biology. No doubt there is a debate on the roles played by Data-driven and Hypothesys-driven research, but just like we do in our everyday practice, many other researchers in System Biology believe that key to reconcile such views on science –that actually are not at all new– within a useful and well-structured framework is *integration*.

As in molecular Biology, explanations in Systems Biology are mechanistic. Following Brigandt [13, 19] we believe that the notion of *explanatory relevance* is primal criteria for integration, one that might be useful beyond mathematical and mechanistic models integration. In our case, *explanatory relevance* is the criteria –or at least one of them– for developing and *enriching* mechanistic models. We exemplified our ideas discussing some work done in our laboratory regarding system’s level modeling of transcriptional regulatory networks in cancer and its relation with metabolic deregulation. Such work integrated information from Data-driven and Hypothesis-driven research coming from several sources and theoretical tools (i.e., complex networks theory and non-equilibrium thermodinamical models) into what we called *enriched mechanistic models*. Our view of enrichment by integration requires that any attempt to add a component to the mechanistic model –being from the description level above or below the actual mechanism– must make an explanatory difference compared to a version of the model that lacks that component.

The idea of *enriched mechanistic models* is just our first sketch of how models are being generated in Systems Biology and how integration is occuring. We hope the notion can shed some light to the study of contemporary Biology practices. We also hold that further studies involving interdisciplinary teams working both in science and in philosophy and sociology of science will prove extremely useful with view of having a better understanding (both at the operative and the epistemical level) of how to integrate data and hypotheses in order to produce intelligible models in Systems Biology.
